# Double-Balloon Kyphoplasty Results in Better Radiographic Outcomes Than a Single-Balloon Kyphoplasty in Treating Osteo-Porotic Spinal Fractures

**DOI:** 10.3390/jcm11123407

**Published:** 2022-06-14

**Authors:** Raphael Lotan, Yaron Haimovich, Louis Schorr, Adam Lee Goldstein, Oded Hershkovich

**Affiliations:** 1 Department of Orthopedic Surgery, Wolfson Medical Center, Affiliated to the Faculty of Medicine, Tel Aviv University, Ha-Lokhamim St. 62, Holon 5822012, Israel; dr.lotan@gmail.com (R.L.); yaronh@wmc.gov.il (Y.H.); louiss@wmc.gov.il (L.S.); 2 Trauma Unit, Wolfson Medical Center, Affiliated to the Faculty of Medicine, Tel Aviv University, Holon 5822012, Israel; adamg.barefoot@gmail.com

**Keywords:** osteoporotic fractures, vertebral compression fractures, balloon kyphoplasty

## Abstract

Background: Studies have found that unilateral and bilateral kyphoplasty have comparable clinical outcomes. Only a few studies have compared the radiographic results of using unilateral vs. simultaneous bilateral approaches. We aimed to examine and compare the radiographic results of unilateral (UKP) vs. bilateral simultaneous double-balloon kyphoplasty (DKP) for treating symptomatic vertebral compression fractures (VCF). Methods: A retrospective cohort of all patients treated for VCF by DKP and UKP over five years in a single medical center. From 2009 to 2012, we routinely performed UKP; from 2012, DKP was the routine due to potential benefits in vertebral realignment. We evaluated pre- and post-surgical fracture characteristics including vertebral height, sagittal and coronal Cobb angle, and fracture reduction. Statistical analysis included a *t*-test for independent variables and Pearson’s correlation. Results: The study cohort consisted of 81 patients (75.8 years ± 10.86) who underwent surgery, with a total of 119 vertebras. We performed 89 UKP on fractured vertebras and 30 DKP on 30 vertebrae. The UKP average fluoroscopy radiation exposure was 15.8 mGy (±11.5) per level compared to 11.2 mGy (±8.7) for DKP, *p* = 0.03. DKP showed significant fracture reduction, 2.8 degrees of Cobb angle, equaling the patient positioning effect on fracture reduction. Conclusion: DKP results in better fracture reduction than UKP, and equals the effect of patient positioning without increased radiation exposure or adverse events.

## 1. Introduction

Osteoporotic vertebral compression fractures (VCFs) are common in the aging population, especially women, accounting for over a million cases each year [1,2]. The prevalence of osteoporosis in the world is rising. The National Osteoporosis Foundation released updated prevalence data estimating that approximately 9 million adults in the United States have osteoporosis. An additional 43 million have low bone mass, which places all of them at increased risk for osteoporosis and fractures [3].

VCFs cause significant acute and chronic morbidity, and fractures may result in functional limitations, persistent pain, loss of independence, and respiratory distress [4,5]. VCFs cause intractable pain, contribute to kyphosis, and significantly reduce patients’ life quality. The vicious cycle starts with a VCF causing a kyphotic deformity, leading to chronic back pain due to biomechanical load changes, higher susceptibility to fractures, and increased kyphosis, leading to an additional risk of kyphotic deformity and vice versa [6].

Initial treatment of an osteoporotic vertebral compression fracture should include pain control, resumption of activity as quickly as possible and physical therapy [7]. Before applying percutaneous minimally invasive surgery, traditional analgesics and bed rest were the main therapeutic measures. Although two-thirds of these fractures gradually improve with conservative treatment, patients may suffer from intractable pain, decreased self-esteem, senile kyphosis, and mood disorders. Previous studies examined the effect of VCFs on mortality with mixed results [8,9,10,11]. Patients who do not show any timely significant pain relief under conservative treatment or do not tolerate oral analgesics are offered percutaneous vertebroplasty or kyphoplasty [12].

Balloon kyphoplasty (BKP) is commonly used as a minimally invasive surgical treatment for pain reduction, vertebral stabilization, and potential spinal deformity reduction [13]. Most deformity correction is achieved by patient positioning. The BKP itself allows further fracture reduction and alignment maintenance. The BKP commences with inserting and expanding an inflatable balloon bone tamp into the fractured vertebral body, through the pedicles percutaneously. Following the balloon inflation and reduction of the fractured vertebra, the cement, usually polymethylmethacrylate (PMMA), is inserted into the space created by the balloon. Good clinical results and some restoration of the vertebral body height have been reported [14,15,16,17,18,19,20,21]. BKP is frequently used for single and multilevel fractures [22,23,24,25,26,27]. Previous studies have reported that unilateral BKP (UKP) is comparable to bilateral BKP using a single balloon, with satisfactory results and fewer complications. Unilateral BKP showed reduced procedure time, costs, radiation exposure, and the risk associated with additional needle placement and cement leakage [28,29,30,31,32,33,34,35]. Only a few studies have compared simultaneous double-balloon BKP to unilateral BKP [13,36,37].

Our aim in the current study was to examine the radiographic results of unilateral BKP (UKP) with simultaneous bilateral balloon inflation BKP (DKP) in the treatment of VCFs. In theory, the inflation of two balloons simultaneously should improve fracture reduction, cementation volume, and dispersion. We conducted a retrospective single-center study and collected our data over eight years (November 2008 to January 2015).

## 2. Materials and Methods

A single-center retrospective cohort of patients underwent single or multiple-level BKP for VCFs from November 2008 to January 2015. The inclusion criteria for BKP surgery included a VCF diagnosed by radiographs, CT scan, and when needed, by bone scan or MRI. The VCF had to cause intractable pain that had failed conservative treatment for two weeks or precluded patient ambulation immediately. The exclusion criteria included conservative treatment and non-osteoporotic vertebral fractures, such as metastatic or myeloma-induced fractures.

From November 2008 to June 2013, all VCFs requiring surgery underwent UKP. In July 2013, our treatment protocol changed to DKP to potentially improve fracture reduction and PMMA dispersion. Demographic data collected included: age, gender, level of fractured vertebrae, and fracture-to-BKP time interval. Ionizing radiation exposure was measured as mGy (Milli-Gray units) per level treated.

Two North American fellowship-trained spine surgeons performed all BKPs. Each BKP was performed under general anesthesia. Two grams of Cefazolin, or 600 mg of Clindamycin when penicillin or cephalosporin allergy was present, was administered intravenously 30 min before surgery. Patients were placed in a prone position. Following draping, one 0.5 cm skin incision was made in the UKP group under fluoroscopy over the pedicle of the fractured vertebra and two incisions were made in the DKP group. Two 13-gauge bone trocars were placed through one or two pedicles under fluoroscopic guidance. A 20 mm inflatable balloon was introduced through the trocars (Kyphon, Medtronic, Minneapolis, MN, USA) and inflated under fluoroscopic control. A single balloon was used and kept inflated for at least 7 min in the UKP group while PMMA was prepared to a toothpaste-like viscosity. In the DKP group, two 20 mm balloons were inflated simultaneously for the same amount of time. Then, the balloons were deflated and retracted. Around 2 to 5 mL of poly-methyl_metacrylate (PMMA) was injected through each trocar under fluoroscopic monitoring of PMMA spread. Each patient was maintained in a supine position for 60 min in the recovery room prior to returning to the orthopedic ward.

Two blinded fellowship-trained spinal surgeons assessed radiographic evaluation of patient radiographs, computerized tomography (CT) scans, and operative fluoroscopic images. The radiographic parameters collected included the posterior and anterior vertebral wall height of the fractured vertebra and a vertebra cephalad and caudal to it, the anterior and posterior wall compression percentage compared to the uninvolved vertebrae, and the sagittal Cobb angle of the fractured segment. Pre-, intra-, and post-surgical parameters were examined.

Statistical analysis included a *t*-test and Levene’s test for independent numerical variables for all the radiographic parameters mentioned. The influence of the time interval between the fracture date and BKP on the radiographic parameters was evaluated using Pearson’s correlation.

## 3. Results

The study cohort consists of 81 patients, 30 males and 51 females, with an average age of 75.8 years (±10.9). One hundred and nineteen vertebras underwent BKP; the male average for fractured vertebras per person was 1.33, and the female average was 1.55 per person. UKP was performed on 89 fractured vertebras, while DKP was performed on 30 fractured vertebras.

The UKP average fluoroscopy radiation exposure was 15.8 mGy (±11.5) per level. DKP fluoroscopy radiation exposure was 11.2 mGy (±8.7) per level. The fluoroscopic exposure difference between groups was statistically significant (*p* = 0.03), favoring DKP.

The measured preoperative kyphosis was 14.3 degrees in the UKP group and 12.3 degrees in the DKP group (*p* = 0.05). Both groups had similar preoperative anterior and posterior percentage of wall compression (Table 1). On prone positioning, the kyphosis correction was 11 degrees for both techniques (Table 1), creating an equal starting point for both groups (*p* = 0.774).

The post-procedural Cobb angle improved significantly for DKP compared to UKP, that is, 4.50 vs. 1.60, respectively (*p* = 0.007). Other radiographic parameters, and restoration of anterior and posterior vertebral walls did not differ between UKP and DKP (Table 1 and Table 2). DKP showed a better anterior wall height restoration trend but did not reach statistical significance.

There were no adverse clinical events in the UKP and DKP groups, such as post-surgical neurological injury, infection, and PMMA leakage requiring revision. We recorded two cases of adjacent level fractures within three months of the index surgery, one in each group.

## 4. Discussion

Kyphoplasty is a viable, widespread treatment option for vertebral compression fractures. There is uncertainty regarding the clinical and radiographic outcomes of BKP and whether changing the surgical technique improves those outcomes. There is variability in BKP techniques; they can be performed unilaterally, bilaterally with a single balloon, or using two balloons simultaneously. The double-balloon technique has the potential benefit of improved leverage over the vertebral endplates, thus improving fracture reduction and a larger space is created for PMMA injection. This technique modification was published previously but did not gain wide acceptance, probably due to a small cohort and conflicting results regarding its advantages [13,32,36]. Based on those preliminary results and the potential benefits, we decided to examine UKP and DKP radiographic outcomes.

The UKP and DKP groups were statistically comparable regarding age, gender, fracture age at surgery, initial anterior and posterior wall compression, and post-positioning Cobb angle. There was a 2-degree difference in preoperative kyphosis (14.3 degrees in the UKP group and 12.3 degrees in the DKP group), presenting a trend that did not reach statistical significance (*p* = 0.05). Despite this initial minor difference, the post positioning Cobb angle was the same (10.9 and 11 degrees, respectively), which represented an equal starting point for both techniques.

DKP showed better fracture reduction, with a 2.80-degree difference, than UKP (*p* = 0.007). The Wang et al. study showed a 0.80 difference in post-procedural fracture reduction using a similar method. However, this study’s main limitation was the small cohort size (48 patients) and the lack of documentation regarding the positioning effect on fracture reduction [36]. In our study, patient positioning was important for fracture reduction as it improved the Cobb angle by 1.3–3.3 degrees (Table 1). Our findings support previous publications on the role of positioning and the comparable results of BKP and vertebroplasty [38,39]. On the other hand, in our study, DKP showed the same additional value to fracture reduction as positioning (4.51 degrees) and was superior to UKP (1.68 degrees). This is the first study to describe such a significant difference in fracture reduction using DKP and comparing it to the effect of patient positioning, which is known for its powerful impact on fracture reduction.

Clinically relevant adverse effects, such as infection, neurological injury, or PMMA leakage requiring revision surgery or causing radicular pain, were not found in the DKP and UKP groups.

An unexpected difference between DKP and UKP was the radiation exposure per level; the UKP group was exposed to 4.6 mGy more per level than the DKP group. This difference could well be attributed to the surgeon’s experience over time and not the technique. The fluoroscopy machines used were not replaced over the years, so hardware improvement was not applicable. Another factor that could explain this anomaly is that DKP was performed simultaneously by two skilled and experienced spine surgeons. Our radiation exposure was comparable and even lower than values published previously [40,41], thus reducing concerns of higher radiation exposure using DKP.

Our main study limitations include its retrospective character and medium-sized cohort. Chronological patient inclusion might have influenced the results towards DKP through surgical experience gained over time. Another limitation is the lack of clinical data, as only radiographic parameters were appraised. Larger cohorts are needed to fully assess the difference in adverse effects between UKP and DKP, as they are rare occurrences.

## 5. Conclusions

In conclusion, DKP results in better fracture reduction than UKP, equaling the effect of patient positioning without increased radiation exposure or adverse events. Further large studies should assess the clinical value of the improved fracture reduction achieved by DKP and whether it is cost-effective, given the additional financial burden of DKP over UKP.

## Figures and Tables

**Table 1:** Balloon kyphoplasty radiographic parameters (average).

	UKP	SD	DKP	SD	*p*-Value
Cobb angle on presentation	14.3°	±7.4°	12.3°	±6.1°	0.20
Cobb angle on positioning	10.9°	±7.2°	11.0°	±5.9°	0.95
Cobb angle following procedure	9.3°	±6.3°	6.5°	±3.9°	0.03
Initial Posterior wall compression %	9.1	17.1±	11.5	±12.3	0.49
Initial Anterior wall compression %	29.2	24.3±	31.1	16.5±	0.72
Post-BKP Posterior wall compression %	4.0	20.3±	5.1	±10.3	0.79
Post-BKP Anterior wall compression %	14.1	±28.2	10.4	16.0±	0.50

Abbreviations: Balloon Kyphoplasty = BKP; Unilateral Balloon Kyphoplasty (UKP); Bilateral Simultaneous Double-Balloon Kyphoplasty (DKP); SD = standard deviation.

**Table 2:** Radiographic differences in UKP versus DKP.

		Levene’s Test For Equality of Variances		*t*-Test for Equality of Means		
		F	Sig	t	df	Sig (2-Tailed)
Fracture Age	Equal variances assumed	0.781	0.379	1.310	0.115	0.193
	Equal variances not assumed			1.508	58.913	0.137
Posterior wall compression % of change	Equal variances assumed	0.4698	0.032	0.305	0.116	0.761
	Equal variances not assumed			0.364	68.002	0.717
Anterior wall compression % of change	Equal variances assumed	0.3785	0.054	0.101	0.116	0.920
	Equal variances not assumed			0.137	94.155	0.891
Cobb angle on positioning	Equal variances assumed	3.089	0.081	0.288	0.116	0.774
	Equal variances not assumed			0.345	68.401	0.731
Cobb angle change following BKP	Equal variances assumed	5.654	0.02	2.20	114	0.03
	Equal variances not assumed			2.78	79	0.007

Abbreviations: Balloon Kyphoplasty = BKP; Unilateral Balloon Kyphoplasty (UKP); Bilateral Simultaneous Double-Balloon Kyphoplasty (DKP).

## Abbreviations

Osteoporotic Vertebral Compression Fractures = VCF; Balloon Kyphoplasty = BKP; Unilateral Balloon Kyphoplasty (UKP); Bilateral Simultaneous Double-Balloon Kyphoplasty (DKP).

## References

[1] Johnell O., Kanis J.A. (2006). An estimate of the worldwide prevalence and disability associated with osteoporotic fractures. Osteoporos. Int..

[2] Rupp M., Walter N., Pfeifer C., Lang S., Kerschbaum M., Krutsch W., Baumann F., Alt V. (2021). The incidence of fractures among the adult population of Germany: An analysis from 2009 through 2019. Dtsch. Ärzteblatt Int..

[3] Huang Z., Wan S., Ning L., Han S. (2014). Is unilateral kyphoplasty as effective and safe as bilateral kyphoplasties for osteoporotic vertebral compression fractures? A meta-analysis. Clin. Orthop. Relat. Res..

[4] Ensrud K.E., Thompson D.E., Cauley J.A., Nevitt M.C., Kado D.M., Hochberg M.C., Santora A.C., Black D.M. (2000). Prevalent vertebral deformities predict mortality and hospitalization in older women with low bone mass. J. Am. Geriatr. Soc..

[5] Schlaich C., Minne H., Bruckner T., Wagner G., Gebest H., Grunze M., Ziegler R., Leidig-Bruckner G. (1998). Reduced pulmonary function in patients with spinal osteoporotic fractures. Osteoporos. Int..

[6] Rostom S., Allali F., Bennani L., Abouqal R., Hajjaj-Hassouni N. (2012). The prevalence of vertebral fractures and health-related quality of life in postmenopausal women. Rheumatol. Int..

[7] Agulnek A.N., O’Leary K.J., Edwards B.J. (2009). Acute vertebral fracture. J. Hosp. Med. Off. Publ. Soc. Hosp. Med..

[8] Rousing R., Hansen K.L., Andersen M.O., Jespersen S.M., Thomsen K., Lauritsen J.M. (2010). Twelve-months follow-up in forty-nine patients with acute/semiacute osteoporotic vertebral fractures treated conservatively or with percutaneous vertebroplasty: A clinical randomized study. Spine.

[9] Kallmes D.F., Comstock B.A., Heagerty P.J., Turner J.A., Wilson D.J., Diamond T.H., Edwards R., Gray L.A., Stout L., Owen S. (2009). A randomized trial of vertebroplasty for osteoporotic spinal fractures. N. Engl. J. Med..

[10] Buchbinder R., Osborne R.H., Ebeling P.R., Wark J.D., Mitchell P., Wriedt C., Graves S., Staples M.P., Murphy B. (2009). A randomized trial of vertebroplasty for painful osteoporotic vertebral fractures. N. Engl. J. Med..

[11] Lotan R., Smorgick Y., Anekstein Y., Rudik O., Prosso I., Hershkovich O. (2021). Kyphoplasty for Elderly Patients with Vertebral Compression Fractures—Do We Save Lives? Mortality Rates Analysis Comparison in a Long-Term Follow-Up Cohort. Glob. Spine J..

[12] Guan H., Yang H., Mei X., Liu T., Guo J. (2012). Early or delayed operation, which is more optimal for kyphoplasty? A retrospective study on cement leakage during kyphoplasty. Injury.

[13] Garfin S.R., Buckley R.A., Ledlie J. (2006). Balloon kyphoplasty for symptomatic vertebral body compression fractures results in rapid, significant, and sustained improvements in back pain, function, and quality of life for elderly patients. Spine.

[14] Jarvik J.G. (2009). Point of view. Efficacy of vertebroplasty and kyphoplasty. Spine.

[15] McGirt M.J., Parker S.L., Wolinsky J.-P., Witham T.F., Bydon A., Gokaslan Z.L. (2009). Vertebroplasty and kyphoplasty for the treatment of vertebral compression fractures: An evidenced-based review of the literature. Spine J..

[16] Wardlaw D., Cummings S.R., Van Meirhaeghe J., Bastian L., Tillman J.B., Ranstam J., Eastell R., Shabe P., Talmadge K., Boonen S. (2009). Efficacy and safety of balloon kyphoplasty compared with non-surgical care for vertebral compression fracture (FREE): A randomised controlled trial. Lancet.

[17] Papadopoulos E.C., Edobor-Osula F., Gardner M.J., Shindle M.K., Lane J.M. (2008). Unipedicular balloon kyphoplasty for the treatment of osteoporotic vertebral compression fractures: Early results. J. Spinal Disord. Tech..

[18] Coumans J.-V.C., Reinhardt M.-K., Lieberman I.H. (2003). Kyphoplasty for vertebral compression fractures: 1-year clinical outcomes from a prospective study. J. Neurosurg. Spine.

[19] Berlemann U., Franz T., Orler R., Heini P.F. (2004). Kyphoplasty for treatment of osteoporotic vertebral fractures: A prospective non-randomized study. Eur. Spine J..

[20] Grohs J.G., Matzner M., Trieb K., Krepler P. (2005). Minimal invasive stabilization of osteoporotic vertebral fractures: A prospective nonrandomized comparison of vertebroplasty and balloon kyphoplasty. Clin. Spine Surg..

[21] Hulme P.A., Krebs J., Ferguson S.J., Berlemann U. (2006). Vertebroplasty and kyphoplasty: A systematic review of 69 clinical studies. Spine.

[22] Lieberman I., Dudeney S., Reinhardt M.-K., Bell G. (2001). Initial outcome and efficacy of "kyphoplasty" in the treatment of painful osteoporotic vertebral compression fractures. Spine.

[23] Phillips F.M., Ho E., Campbell-Hupp M., McNally T., Wetzel F.T., Gupta P. (2003). Early radiographic and clinical results of balloon kyphoplasty for the treatment of osteoporotic vertebral compression fractures. Spine.

[24] Ledlie J.T., Renfro M.B. (2006). Kyphoplasty treatment of vertebral fractures: 2-year outcomes show sustained benefits. Spine.

[25] Voggenreiter G. (2005). Balloon kyphoplasty is effective in deformity correction of osteoporotic vertebral compression fractures. Spine.

[26] Pradhan B.B., Bae H.W., Kropf M.A., Patel V.V., Delamarter R.B. (2006). Kyphoplasty reduction of osteoporotic vertebral compression fractures: Correction of local kyphosis versus overall sagittal alignment. Spine.

[27] Singh A.K., Pilgram T.K., Gilula L.A. (2006). Osteoporotic compression fractures: Outcomes after single-versus multiple-level percutaneous vertebroplasty. Radiology.

[28] Chen L., Yang H., Tang T. (2011). Unilateral versus bilateral balloon kyphoplasty for multilevel osteoporotic vertebral compression fractures: A prospective study. Spine.

[29] Hu M.M., Eskey C.J., Tong S.C., Nogueira R.G., Pomerantz S.R., Rabinov J.D., Pryor J.C., Hirsch J.A. (2005). Kyphoplasty for vertebral compression fracture via a uni-pedicular approach. Pain Physician.

[30] Chang W.S., Lee S.-H., Choi W.G., Choi G., Jo B.J. (2007). Unipedicular vertebroplasty for osteoporotic compression fracture using an individualized needle insertion angle. Clin. J. Pain.

[31] Hoh B.L., Rabinov J.D., Pryor J.C., Hirsch J.A. (2004). Balloon kyphoplasty for vertebral compression fracture using a unilateral balloon tamp via a uni-pedicular approach. Pain Physician.

[32] Rebolledo B., Gladnick B., Unnanuntana A., Nguyen J., Kepler C., Lane J.M. (2013). Comparison of unipedicular and bipedicular balloon kyphoplasty for the treatment of osteoporotic vertebral compression fractures: A prospective randomised study. Bone Jt. J..

[33] Xiang G.-H., Tong M.-J., Lou C., Zhu S.-P., Guo W.-J., Ke C.-R. (2018). The role of unilateral balloon kyphoplasty for the treatment of patients with OVCFS: A systematic review and meta-analysis. Pain Physician.

[34] Tan G., Li F., Zhou D., Cai X., Huang Y., Liu F. (2018). Unilateral versus bilateral percutaneous balloon kyphoplasty for osteoporotic vertebral compression fractures: A systematic review of overlapping meta-analyses. Medicine.

[35] Zhang F., Zhao Q.-M., Ni X.-H., Wang L.-J., Ma Z.-G., Kang P., Liu X.-D., Yin S. (2021). Comparison of unilateral and bilateral puncture percutaneous kyphoplasty in the treatment of osteoporotic vertebral compression fractures. Neurosci. J..

[36] Wang H., Sun Z., Wang Z., Jiang W. (2015). Single-balloon versus double-balloon bipedicular kyphoplasty for osteoporotic vertebral compression fractures. J. Clin. Neurosci..

[37] Jing Z., Dong J., Li Z., Nan F. (2018). Single balloon versus double balloon bipedicular kyphoplasty: A systematic review and meta-analysis. Eur. Spine J..

[38] Lee S.-T., Chen J.-F. (2004). Closed reduction vertebroplasty for the treatment of osteoporotic vertebral compression fractures. J. Neurosurg. Spine.

[39] Chin D.-K., Kim Y.-S., Cho Y.-E., Shin J.-J. (2006). Efficacy of postural reduction in osteoporotic vertebral compression fractures followed by percutaneous vertebroplasty. Neurosurgery.

[40] Mroz T.E., Yamashita T., Davros W.J., Lieberman I.H. (2008). Radiation exposure to the surgeon and the patient during kyphoplasty. Clin. Spine Surg..

[41] Synowitz M., Kiwit J. (2006). Surgeon’s radiation exposure during percutaneous vertebroplasty. J. Neurosurg. Spine.

